# Investigating melanogenesis-related microRNAs as disease biomarkers in vitiligo

**DOI:** 10.1038/s41598-022-17770-3

**Published:** 2022-08-08

**Authors:** Hoda Y. Abdallah, Noura R. Abdelhamid, Eman A. Mohammed, Nehal Y. AbdElWahab, Noha Z. Tawfik, Amal H. A. Gomaa, Eman A. Toraih, Alia Ellawindy

**Affiliations:** 1 Medical Genetics Unit, Department of Histology and Cell Biology, Faculty of Medicine, Suez Canal University, Ismailia, 41522 Egypt; 2 Center of Excellence in Molecular and Cellular Medicine, Faculty of Medicine, Suez Canal University, Ismailia, 41522 Egypt; 3 Dermatology, Venereology and Andrology Department, Faculty of Medicine, Suez Canal University, Ismailia, 41522 Egypt; 4 Division of Endocrine and Oncologic Surgery, Department of Surgery, School of Medicine, Tulane University, New Orleans, LA 70112 USA

**Keywords:** Genetics, Molecular biology, Biomarkers, Diseases, Molecular medicine

## Abstract

Vitiligo is considered a disabling disease that affects physical, social, psychological, and occupational aspects of an individual's quality of life. The search for non-invasive and reliable biomarkers for vitiligo's early diagnosis, prognosis, and treatment prediction is under intensive investigation. There is currently an emerging interest in employing miRNAs as biomarkers to predict vitiligo diagnosis and prognosis, inspired by the well-preserved nature of miRNAs in serum or plasma. In the current study, we assessed a panel of 20 melanogenesis pathway-related microRNAs (miRNAs) using quantitative real-time PCR technique in 85 non-segmental vitiligo (NSV) patients compared to 85 normal controls followed by function and pathway enrichment analysis for the miRNAs with significant results. Twelve out of the 20 circulating miRNAs showed significantly higher expression levels in vitiligo patients relative to controls where miR-423 show the highest expression level followed by miR-182, miR-106a, miR-23b, miR-9, miR-124, miR-130a, miR-203a, miR-181, miR-152, and miR-320a. While six miRNAs (miR-224, miR-148a, miR-137, and miR-7, miR-148b, miR-145, miR-374b, and miR-196b) didn’t show significant expression level. The analysis of the receiver operating curve indicated that miR-423, miR-106a, and miR-182 were outstanding biomarkers with the highest areas under the curve in vitiligo. This study is the first Egyptian study to investigate a panel of miRNAs expression profile in the plasma of patients with NSV. Our results suggest that specific circulating miRNAs signature might be implicated in vitiligo pathogenesis and could potentially be used as biomarkers in vitiligo.

## Introduction

Vitiligo is a common, acquired discoloration of the skin affecting about 1–4% of the world population, presenting as milky-white patches over the skin and/or mucosa. Although the disease does not produce direct physical impairment, it may considerably influence the psychological well-being of the patients^1,2^, resulting in psychosocial distress and social stigmatization. So, vitiligo is considered a disabling disease that affects physical, social, psychological, and occupational aspects of an individual's quality of life (QoL).

Vitiligo can be classified into two major types; segmental vitiligo (SV) and non-segmental vitiligo (NSV), which accounts for nearly 90% of total vitiligo cases^3^. Vitiligo usually appears in childhood or early adulthood, with the highest incidence between 10 to 30 years^4^.

Although vitiligo has been known for thousands of years—only recently—real progress in understanding its molecular and pathological basis has emerged, which may hopefully facilitate progress in vitiligo treatment and ultimately even prevention^5^. The most supported etiology is the autoimmune hypothesis^6^. Autoantibodies against melanocytes were detected in patients' sera and have recently been used to predict disease prognosis^7^. Association of other autoimmune diseases, such as Hashimoto's thyroiditis, Grave's disease, and pernicious anemia with vitiligo, reinforce the autoimmune cause^8^. Besides immune cause, toxic damage to melanocytes due to metabolic derangements are gaining support with time^9^.

In all cases, vitiligo is a polygenic disease entailing complex interaction between genetic and non-genetic factors^10,11^. Among the genetic factors that could be implicated in the pathogenesis of vitiligo; are the microRNAs (miRNAs). MiRNAs are short (19–25 nucleotides) non-coding RNAs (ncRNAs) that are involved in the regulation of gene expression at both the post-transcriptional and translation level through imperfect binding with the 3′ untranslated region (3′UTR) of the target mRNA and subsequent degradation of mRNA or translational repression^3,12–14^. Multiple miRNAs can target a single mRNA molecule, while one miRNA can interact with multiple mRNAs^15^.

Various human miRNAs have been widely investigated in the past ten years. They have been found to regulate diverse physiological processes, including cell proliferation, differentiation, development, signal transduction, metabolism, apoptosis, and immune responses. Their abnormal expression is involved in developing many diseases^16^. It has been demonstrated that miRNAs circulate in a highly stable cell-free form in various body fluids including plasma, serum, saliva, milk, and urine, and are believed to be promising biomarkers for different diseases^17^.

Many studies have demonstrated the crucial role of miRNAs on the development, proliferation, and survival of cells, including the immune cells and melanocytes^3,12,13,18^. Deregulation of miRNAs was the underlying pathology in many inflammatory skin disorders such as atopic dermatitis, allergic contact dermatitis, and psoriasis^19^. Microarray analysis has elucidated the abnormal expression of multiple miRNAs in the skin and serum of patients with vitiligo^12^. Currently, there is an emerging interest in employing miRNAs as biomarkers to predict disease prognosis and response to treatment, inspired by the well-preserved nature of miRNAs in serum or plasma^15^.

In this vicinity, we have done this pilot study to investigate the differential expression of a panel of 20-miRNAs in the plasma collected from age-matched vitiligo patients and healthy controls using quantitative real-time PCR (qRT-PCR). Those 20-miRNAs are involved in the melanogenesis pathway, have putative binding sites for the most affected genes in vitiligo, and were selected using bioinformatics databases.

## Subjects and methods

### Study participants

The present study included one hundred and seventy participants. The study subjects were divided into two groups; (1) Study Group: 85 adult patients from both genders diagnosed with vitiligo by clinical examination and Woods's lamp recruited from the Dermatology outpatient clinic, Suez Canal University (SCU) Hospital, Ismailia, Egypt, and (2) Control Group: 85 healthy non-related participants, matched by age and gender to the study group. The clinicopathological data, including patients' age, sex, BMI, family history, past history of other autoimmune diseases (e.g., diabetes mellitus, Hashimoto's thyroiditis, Addison's disease, psoriasis), age of disease onset, disease duration, severity, and treatment were collected from the patients' history. All patients were subjected to detailed dermatological examination to determine: the site, size, pattern, and distribution of individual lesions, assessment of disease severity was performed according to the criteria of the vitiligo area severity index (VASI), vitiligo disease activity score (VADI) and the Vitiligo European Task Force (VETF) (Kawakami and Hashimoto, 2011).

### Selection of the 20 circulating miRNAs understudy using bioinformatics tools

The 20 circulating miRNAs involved in the melanogenesis pathway [hsa04916] were selected using online bioinformatics tools, which are DIANA-miRPath web server (http://snf-515788.vm.okeanos.grnet.gr)^20^ and microrna.org^21^ and are listed in Table 1.

**Table 1:** Primer Sequences of circulating miRNAs understudy.

MiRNA	Primer sequence
MiR-7-5p	TGGAAGACTAGTGATTTTG
MiR-9-5p	TCTTTGGTTATCTAGCTGTAT
MiR-23b-5p	CTCCCCAGCATCTTCGATCC
MiR-106a-5p	TGCTTACAGTGCAGGTAG
MiR-124-3p	TTCACAGCGGACCTTGA
MiR-130a-5p	CACATTGTGCTACTGTCT
MiR-137	ATTGCTTAAGAATACGCGT
MiR-145-5p	GTCCAGTTTTCCCAGGA
MiR-148a-5p	GTTCTGAGACACTCCGA
MiR-148b-5p	GTTCTGTTATACACTCAGG
MiR-152	TCAGTGCATGACAGAACT
MiR-155-5p	TGCTAATCGTGATAGGGG
MiR-181a-5p	AACATTCAACGCTGTCGGTG
MiR-182-5p	GGCAATGGTAGAACTCAC
MiR-196b-5p	GGTAGTTTCCTGTTGTTG
MiR-203a-5p	TAGTGGTCCTAAACATTTCAC
MiR-224-5p	CAAGTCACTAGTGGTTCC
MiR-320a-5p	AAGCTGGGTTGAGAGGG
MiR-374b-5p	TATAATACAACCTGATAAGTG
MiR-423-5p	GGGCAGAGAGCGAGAC
SNORD68	GCCCCTGCGCAAGGATGAC
RNU6B	GCCCCTGCGCAAGGATGAC

### Samples collection and total RNA extraction, including small RNA

Three ml of fresh venous blood was collected in vacutainer tubes containing ethylene diamine tetra-acetic acid (EDTA) anticoagulant. Blood samples were centrifuged to separate plasma; 100 μl plasma was preserved in a 500 μl Qiazole reagent. The plasma samples were stored at − 80℃ till further analysis. The total RNA, including small RNA, was isolated from plasma using Qiagen miRNeasy mini kit and (Qiagen, Catalog no. 217004) following the protocol supplied by the manufacturer. An Eppendorf 5417C cooling microcentrifuge with adjusted temperature was used throughout the RNA extraction process. RNA purity and concentration were assessed using the NanoDrop 2000 1C at 260 and 280 nm absorbance (NanoDrop Tech., Inc. Wilmington, DE, USA). A ratio between 1.8 and 2.2 was considered acceptable for further genetic analysis. The wavelength-dependent extinction coefficient "33" was adjusted to represent the micro-component of all RNA in solution.

### The circulating miRNAs relative gene expression quantification using Real-time PCR

The total RNA extracted was subjected to reverse transcription (RT) where complementary DNA (cDNA) was generated from total RNA containing miRNA with the miScript II RT Kit (Qiagen, Catalog no. 218161), in which miRNAs were polyadenylated by poly(A) polymerase and converted into cDNA by reverse transcriptase with oligo-dT priming. RT was carried out in a Veriti™ 96-Well Thermal Cycler (Applied Biosystems, USA) at 37 °C for 1 h, followed by inactivation of the reaction by briefly incubating at 95 °C.

Circulating miRNAs expression profiling was carried out using SYBR Green-based real-time PCR. The premix of cDNA was used as a template for qRT-PCR miRNA expression. Primers for the 20 miRNAs (miR-7, miR-9, miR-23b, miR-106a, miR124, miR-130a, miR-137, miR1-45, miR-148a, miR-148b, miR-152, miR-155, miR-181a, miR-182, miR-196b, miR-203a, miR-224, miR-320a, miR-374b, miR-423) are described in Table 1, and miScript SYBR Green PCR Kit (Qiagen, cat. no 218076) was used to measure the expression levels with a universal reverse primer. RNU6B and SNORD68 were used as endogenous controls to enable data analysis using the ΔΔCT method of relative quantification. The expression levels were done according to the minimum information for publication of quantitative real-time PCR experiments (MIQE) guidelines. Duplicate reactions were placed in each run, and a "no reverse transcribed" controls and a well free template were included in each run. Each plate run initially at 95 °C for 5 min, followed by 40 cycles of denaturation, annealing and extension at 95 °C (15 s), 55 °C (1 min), 72 °C (1 min) respectively.

### Gene expression data analysis

Fold changes for the 20 circulating miRNAs in each patient sample relative to the corresponding control were estimated using Livak method^22^ based on the ΔΔC_q_ method where ΔΔC_q_ = (C_q__Cir-miRNA_ − C_q SNORD68/RNU6B_) _vitiligo_ −  (C_q Cir-miRNA_ − C_q SNORD68/RNU6B_) _control_. Cq stands for quantification (threshold).

### Function and pathway enrichment analysis of the circulating miRNAs understudy

Functional enrichment analysis of the 20 circulating miRNAs understudy was analyzed using gene analytics software (https://geneanalytics.genecards.org)^23^, and gProfiler software (http://biit.cs.ut.ee/gprofiler/gost)^24^. The gene ontology GO terms that matched our miRNAs understudy were presented in the order of the matching scores. The binomial distribution was used to test the null hypothesis that the input biomarkers were not over-represented within any SuperPath or GO term. The presented score for each biomarker is a transformation (log2) of the resulting *p* value, where higher scores indicated better matches. Results with *p* values lower than 10–50 were assigned the maximum score. The pathway enrichment analysis was conducted using the software Database for Annotation Visualization and Integrated Discovery (DAVID) (https://david.ncifcrf.gov/)^25^, and DIANA Tool mirPath v.3 (http://snf-515788.vm.okeanos.grnet.gr/)^20^, where GO consisting of cellular components, biological processes, and molecular functions terms was searched for via pathway analysis on the Kyoto Encyclopedia of Genes and Genomes (KEGG) database, for determining the affected pathways with differential miRNA expression and their target genes.

### MiRNA regulatory network construction

The targets of the statistically significant differentially expressed miRNAs (DEmiRNAs) were predicted using miRTargetLink 2.0 (Version 2.0, https://ccb-compute.cs.uni-saarland.de/)^26^. Statistically homogenous and significant DEGs targets were kept, and the miRNA-mRNA pairs negative correlations were also included.

### Statistical analysis

Statistical Package for the Social Sciences (SPSS) for Windows software version 26.0 (Armonk, NY: IBM Corp.) was used. The study power and the sample size were calculated by G*Power version 3.1.9.2. The given power for gene expression study design option was 85% at total sample size = 170. Continuous variables were presented as means ± standard deviations, while categorical variables were presented as frequencies and percentages. Data profile was tested for outliers and normality. When appropriate, Mann–Whitney and Student t tests were used to compare between cases and controls. The Spearman correlation test was used for determining the correlation coefficient. Spearman's rank test was done for two-sided *p* values correlation analysis. A two-tailed *p* value of < 0.05 was considered statistically significant. The area under the curve (AUC) of receiver operating characteristic (ROC) was plotted to evaluate putative biomarkers' diagnostic and prognostic value.

### Ethics approval and consent to participate

The study was approved by the Suez Canal University, Faculty of Medicine, Ethics Committee in Ismailia, Egypt (Approval No. 4210) and conducted according to the Declaration of Helsinki’s guidelines. Informed consent was obtained from all individual participants included in the study.

## Results

### Baseline characteristics and risk factors among the study population

Table 2 shows the baseline characteristics and risk factors among our study population. The age distribution among the study population ranged from 6 to 93 years. The mean age for the patients was 35.41 ± 17.7, and the mean age for the controls was 35.47 ± 18.75, with no statistically significant difference between both groups. Regarding Special habits, 56.5% of patients were passive smokers, 21.2% smokers, and 22.2% non-smokers. In comparison, 27.1% of controls were passive smokers, 20% smokers, and 37.5% non-smokers, while non-smoking showed statistically significant protective relation against vitiligo. There was no statistical difference among both groups concerning obesity grades. The family history and exposure to stress showed a highly significant correlation among our vitiligo patients.

**Table 2:** Baseline characteristics and risk factors among the study population.

Variable	Cases (n = 85)	Controls (n = 85)	*P* value	Odds ratio (95% CI)
**Age, mean (year)**	35.41 ± 17.7	35.47 ± 18.75	0.98	–
**Age group**				
˂ 40	31 (36.4%)	30 (35.3%)	–	Reference
20–40	35 (41.2%)	33 (38.8%)	0.94	1.0 (0.5–2.0)
˃ 20	19 (22.4%)	22 (25.9%)	0.66	0.8 (0.4–1.8)
**Gender**				
Females	52 (61.2%)	53 (62.4%)	–	Reference
Males	33 (38.8%)	32 (37.6%)	0.87	1.0 (0.6–1.9)
**Smoking**				
Passive	48 (56.5%)	23 (27.1%)	–	Reference
Smoker	18 (21.2%)	17 (20%)	0.1	0.5 (0.2–1.2)
Non-smoker	19 (22.2%)	45 (37.5%)	< 0.0001*	0.2 (0.1–0.4)
**Degree of obesity**				
Normal	23 (27.1%)	25 (29.4%)	–	Reference
Underweight	5 (5.8%)	3 (3.5%)	0.45	1.8 (0.4–8.4)
Overweight	55 (64.7%)	53 (62.4%)	0.73	1.1 (0.6–2.2)
Obese	2 (2.4%)	4 (4.7%)	0.5	0.5 (0.1–3.3)
**Family history**				
Positive	16 (24.4%)		< 0.0001*	
Negative	69 (81.1%)			
**Exposure to stress**				
Yes	58 (68.2%)		< 0.0001*	
No	27 (31.8%)			

Data are shown as number (percentage) or mean ± SD. *P value < 0.05 was considered as statistically significant. *BMI* body mass index.

### Clinicopathological features among vitiligo patients

Table 3 shows the clinicopathological features among vitiligo patients. The patient’s skin type, premature graying of hair, trichrome, hypopigmentation, follicular re-pigmentation, Koebner’s phenomena, VIDA, and VASI score showed high statistically significant relation with vitiligo. At the same time, the duration of the disease did not appear to affect the clinicopathological features in vitiligo among the studied cohort.

**Table 3:** Clinicopathological features among vitiligo patients.

Variable	Cases (n = 85)
**Skin type**	
Type I	1 (1.2%)
Type II	21 (24.7%)
Type III	42 (49.4%)
Type IV	17 (20%)
Type V	4 (4.7%)
**Premature graying of hair**	
No	59 (69.4%)
Yes	26 (30.6%)
**Duration of disease**	
≤ 1 year	14 (16.5%)
1–5 years	24 (28.2%)
> 5–10 years	22 (25.9%)
> 10 years	25 (29.4%)
**Trichrome**	
No	64 (75.3%)
Yes	21 (24.7%)
**Hypopigmentation**	
No	16 (18.8%)
Yes	69 (81.2%)
**Follicular re-pigmentation**	
No	55 (64.7%)
Yes	30 (35.3%)
**Koebner’s phenomena**	
No	16 (18.8%)
Yes	69 (81.2%)
**VIDA**	
Stage -1	33 (39.3%)
Stage 0	3 (3.6%)
Stage 1	11 (13.1%)
Stage 2	16 (19%)
Stage 3	18 (21.4%)
Stage 4	2 (2.4%)
**VASI**	0.5 ± 0.03

Data are shown as number (percentage). *VASI* vitiligo area severity index, *VIDA* vitiligo disease activity.

### The expression signature of melanogenesis pathway circulating miRNAs in vitiligo patients’

**Figure 1:**
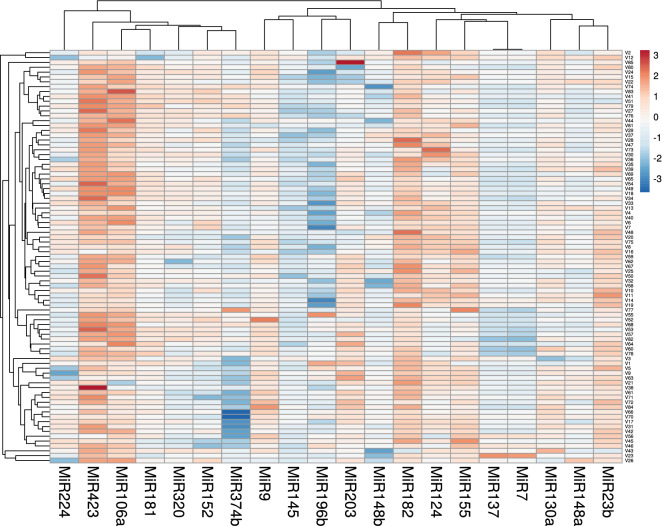
The differential expression profile of circulating miRNAs understudy in vitiligo (n = 85). Heat map illustrates the levels of all miRNAs understudy (Log2fold change) in vitiligo patients. Color grades are shown within each row, with the highest expression corresponding to deep red and the lowest to deep blue.

**Figure 2:**
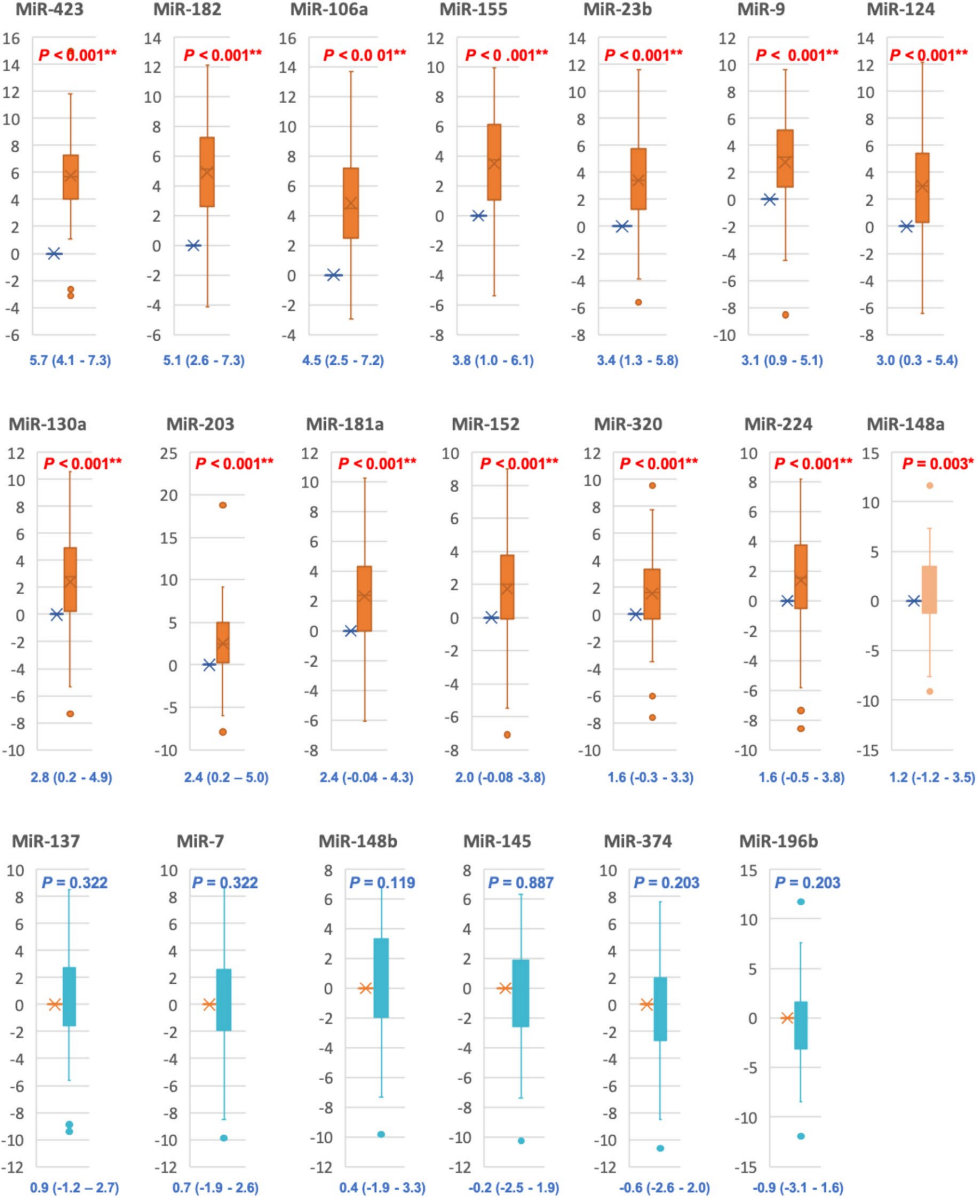
The relative expression level of the circulating miRNAs understudy in vitiligo. Twenty miRNAs were analyzed: miR-7, miR-9, miR-23b, miR-106a, miR124, miR-130a, miR-137, miR1-45, miR-148a, miR-148b, miR-152, miR-155, miR-181a, miR-182, miR-196b, miR-203a, miR-224, miR-320a, miR-374b, and miR-423. SNORD68 and RNU6B were used as endogenous controls. The values are represented as median (Q1 and Q3) using Whiskers and bars. Control level were set at the Log2 fold change equals 0 and all values were log-transformed. Mann–Whitney U test was used for comparison. *Significant at *p* value < 0.05, **Significant at *p* value < 0.01.

As depicted in Figs. 1 and 2, 14 circulating miRNAs showed significantly higher expression levels in vitiligo patients relative to controls where miR-423 show the highest expression level (Log2FC = 5.7) followed by miR-182 (Log2FC = 5.1), miR-106a (Log2FC = 4.5), miR-155 (Log2FC = 3.8), miR-23-b (Log2FC = 3.4), miR-9 (Log2FC = 3.1), miR-124 (Log2FC = 3.0), miR-130a (Log2FC = 2.8), miR-203a (Log2FC = 2.4), miR-181 (Log2FC = 2.4), miR-152 (Log2FC = 2.03), miR-320a (Log2FC = 1.6), miR-224 (Log2FC = 1.6), and miR-148a (Log2FC = 1.2). While miR-137 (Log2FC = 0.9), and miR-7 (Log2FC = 0.7), miR-148b (Log2FC = 0.4), miR-145 (Log2FC = -0.2), miR-374b (Log2FC = -0.5), and miR-196b (Log2FC = -0.9) didn’t show statistically significant expression levels in vitiligo patients relative to controls.

### Circulating miRNAs predictive significance as biomarkers by ROC analysis

The ROC analysis including the AUC and probability levels, were presented in Table 4. The AUCs of the twenty miRNAs ranged from 0.447 to 0.976 are shown in Table 4 indicating miR-423 as the most efficient biomarker with the largest AUC (0.976 at the cutoff point of 1.57-fold; *p* < 0.001, sensitivity = 97.6%, and specificity = 100%), followed by miR-106a (AUC = 0.953), miR-182 (AUC = 0.941) with outstanding discrimination efficiency. MiR-23b (AUC = 0.859), miR-124 and miR-9 (AUC = 0.835) with excellent biomarker discrimination efficiency. MiR-155 (AUC = 0.771), miR-130a (AUC = 0.765), miR-181a (AUC = 0.753), miR-152 (AUC = 0.729), and miR-320a (AUC = 0.706) with acceptable biomarker discrimination power.

**Table 4:** ROC analysis for biomarker accuracy testing of circulating miRNAs understudy.

MiRNA	AUC				
	Area	SE	Asymptotic sig.	Asymptotic 95% CI	
				Lower	Upper
MiR-423	0.976	0.016	< 0.001**	0.944	1.000
MiR-106a	0.953	0.023	< 0.001**	0.908	0.998
MiR-182	0.941	0.026	< 0.001**	0.891	0.991
MiR-23b	0.859	0.038	< 0.001**	0.785	0.933
MiR124	0.835	0.040	< 0.001**	0.756	0.914
MiR-9	0.835	0.040	< 0.001**	0.756	0.914
MiR-155	0.771	0.045	< 0.001**	0.682	0.859
MiR-130a	0.765	0.046	< 0.001**	0.675	0.855
MiR-181	0.753	0.047	< 0.001**	0.661	0.845
MiR-152	0.729	0.048	< 0.001**	0.635	0.824
MiR-320	0.706	0.049	< 0.001**	0.609	0.803
MiR-203a	0.682	0.050	< 0.001**	0.583	0.781
MiR-224	0.671	0.051	< 0.001**	0.571	0.771
MiR-148a	0.624	0.053	0.005*	0.521	0.727
MiR-148b	0.565	0.054	0.145	0.459	0.670
MiR-7	0.541	0.054	0.354	0.435	0.647
MiR-137	0.541	0.054	0.354	0.435	0.647
MiR-145	0.494	0.054	0.895	0.388	0.600
MiR-196b	0.447	0.054	0.233	0.341	0.553
MiR-374b	0.447	0.054	0.233	0.341	0.553

Significant *P* values are in bold. *Significant at *p* value < 0.05, **Significant at *p* value < 0.01. *AUC* area under the curve, *SE* standard error. AUC: 0.5 or less = no discrimination, 0.7–0.8 = acceptable discrimination, 0.8–0.9 = excellent discrimination, and more than 0.9 = outstanding discrimination. *SE* standard error, *Sig* significance.

Significant values are in bold.

### Correlation analysis of circulating miRNAs differential expression levels and vitiligo patients’ clinical characteristics

Concerning clinical characteristics correlations with circulating miRNAs shown in Table 5, skin type showed significant correlations with nearly all miRNAs understudy except for miR-196b, miR-224, and miR-423. While age correlated significantly with miR-203a and VASI score significantly correlated with miR-423 only.

**Table 5:**
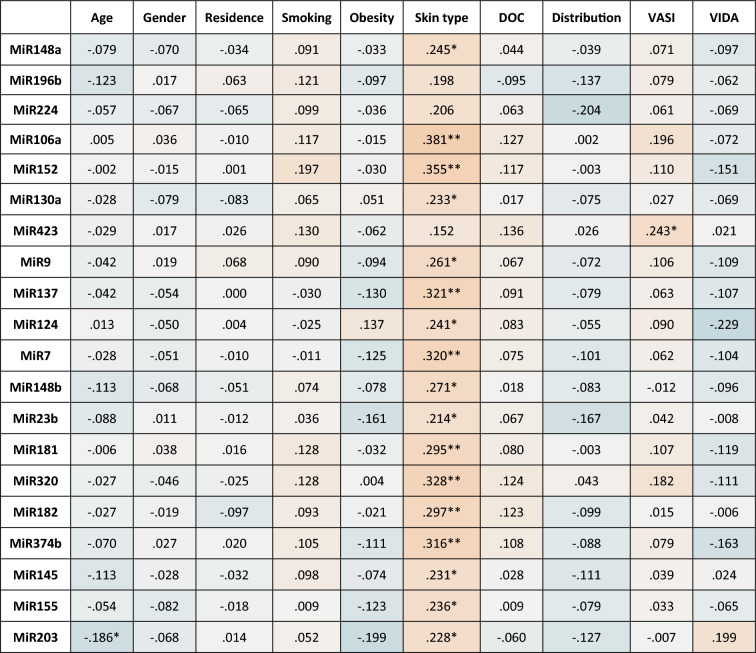
Correlation matrix between miRNAs understudy and clinical characteristics of vitiligo patients.

Spearman’s Correlation coefficient are presented for the 20-miRNAs among vitiligo patients’ understudy. **Correlation is significant at the 0.01 level (2-tailed). *Correlation is significant at the 0.05 level (2-tailed). *DOC* duration of complain, *VASI* vitiligo area severity index, *VIDA* vitiligo disease activity.

On the other hand, the 20 selected circulating miRNAs showed various distributions among all vitiligo patients. The Spearman’s rank correlation of the 20 selected circulating miRNAs in vitiligo patients and controls was evaluated and presented in Table 6. There was a strong association between nearly all miRNAs understudy in vitiligo s with Spearman’s correlation coefficient of 0.59 and more and a two-tailed significance *p* < 0.001.

**Table 6:**
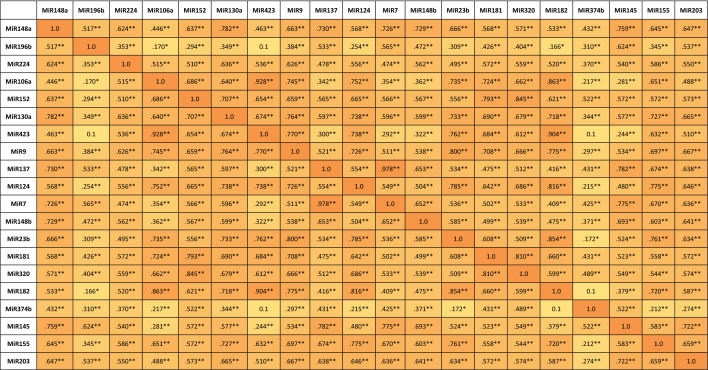
Correlation matrix of the 20 circulating miRNAs understudy.

Spearman’s Correlation coefficient are presented for the 20-miRNAs understudy. Significant values are highlighted. **Correlation is significant at the 0.01 level (2-tailed). *Correlation is significant at the 0.05 level (2-tailed).

At the same time, many correlations were found among our studied risk factors in vitiligo as illustrated in Table 7 where age significantly increased the association with other chronic disease as CAD (r = 0.233, *p* = 0.032), hypertension (r = 0.348, *p* < 0.001), diabetes (r = 0.314, *p* = 0.003) and obesity (r = 0.472, *p* < 0.001). The duration of complaint correlated with skin type (r = 0.302, *p* = 0.005), obesity (r = 0.162, *p* = 0.138) and age (r = 0.425, *p* < 0.001).

**Table 7:** Correlation analysis of vitiligo risk factors in the study.

	CAD	HTN	DM	Stress	FH	DOC	Age	Gender	Smoking	Obesity	Skin type
**CAD**											
r	1	0.451**	0.425**	0.106	0.124	0.199	0.233*	0.040	0.003	0.213	0.090
*P* value	–	0.000	0.000	0.335	0.259	0.068	0.032	0.719	0.980	0.050	0.415
**HTN**											
r	0.451**	1	0.824**	0.153	0.128	0.192	0.348**	0.127	0.238*	0.260*	− 0.010
*P* value	0.000	–	0.000	0.163	0.244	0.078	0.001	0.246	0.028	0.016	0.929
**DM**											
r	0.425**	0.824**	1	0.014	0.104	0.162	0.314**	0.018	0.173	0.270*	0.034
*P* value	0.000	0.000	–	0.900	0.342	0.138	0.003	0.872	0.114	0.013	0.759
**Stress**											
r	0.106	0.153	0.014	1	0.135	− 0.051	0.168	0.113	0.141	0.127	− 0.072
*P* value	0.335	0.163	0.900	–	0.219	0.643	0.125	0.304	0.198	0.248	0.510
**FH**											
r	0.124	0.128	0.104	0.135	1	− 0.083	0.023	− 0.064	0.145	0.040	0.096
*P* value	0.259	0.244	0.342	0.219	–	0.448	0.832	0.563	0.184	0.717	0.384
**DOC**											
r	0.199	0.192	0.162	− 0.051	− 0.083	1	0.425**	0.159	0.160	0.162	0.302**
*P* value	0.068	0.078	0.138	0.643	0.448	–	0.000	0.145	0.144	0.138	0.005
**Age**											
r	0.233*	0.348**	0.314**	0.168	0.023	0.425**	1	0.154	0.187	0.472**	0.084
*P* value	0.032	0.001	0.003	0.125	0.832	0.000	–	0.160	0.086	0.000	0.444
**Gender**											
r	0.040	0.127	0.018	0.113	− 0.064	0.159	0.154	1	0.456**	− 0.206	0.213
*P* value	0.719	0.246	0.872	0.304	0.563	0.145	0.160	–	0.000	0.059	0.050
**Smoking**											
r	0.003	0.238*	0.173	0.141	0.145	0.160	0.187	0.456**	1	0.075	0.260*
*P* value	0.980	0.028	0.114	0.198	0.184	0.144	0.086	0.000	–	0.497	0.016
**Obesity**											
r	0.213	0.260*	0.270*	0.127	0.040	0.162	0.472**	− 0.206	0.075	1	0.017
*P* value	0.050	0.016	0.013	0.248	0.717	0.138	0.000	0.059	0.497	–	0.881
**Skin type**											
r	0.090	− 0.010	0.034	− 0.072	0.096	0.302**	0.084	0.213	0.260*	0.017	1
*P* value	0.415	0.929	0.759	0.510	0.384	0.005	0.444	0.050	0.016	0.881	–

Spearman’s Correlation coefficient are presented for different clinical characteristics among vitiligo patients’ understudy. Significant values are highlighted. **Correlation is significant at the 0.01 level (2-tailed). *Correlation is significant at the 0.05 level (2-tailed). *CAD* coronary artery disease, *DOC* duration of complain, *Distri.* distribution, DM diabetes mellitus, *FH* family history, *HTN* hypertension.

Finally, regarding correlation analysis of the different clinical characteristics of vitiligo in the study shown in Table 8, VASI score significantly correlated with pre-mature graying of hair (r = 0.308, *p* = 0.004), hypopigmentation (r = 0.310, *p* = 0.004), Kopner phenomenon (r = 0.255, *p* = 0.018), and disease distribution (r = 0.421, *p* < 0.001).

**Table 8:** Correlation analysis of vitiligo clinical characteristics in the study.

	Skin type	Pre_Gray	Trichrome	Hypopig	Follicular	Kopner	Distri	VASI	VIDA
**Skin type**									
r	1	− 0.143	− 0.181	− 0.096	0.337**	− 0.096	− 0.046	0.089	− 0.107
*p* value	–	0.193	0.096	0.384	0.002	0.384	0.678	0.419	0.458
**Pre_Gray**									
r	− 0.143	1	− 0.025	0.254*	0.044	0.124	0.081	0.308**	0.032
*p* value	0.193	–	0.820	0.019	0.689	0.259	0.460	0.004	0.823
**Trichrome**									
r	− 0.181	− 0.025	1	0.136	− 0.024	− 0.073	− 0.233*	− 0.143	− 0.076
*p* value	0.096	0.820	–	0.214	0.831	0.506	0.032	0.193	0.599
**Hypopig**									
r	− 0.096	0.254*	0.136	1	0.230*	0.384**	0.087	0.310**	− 0.155
*p* value	0.384	0.019	0.214	–	0.034	0.000	0.430	0.004	0.282
**Follicular**									
r	0.337**	0.044	− 0.024	0.230*	1	− 0.022	− 0.195	0.145	− 0.199
*p* value	0.002	0.689	0.831	0.034	–	0.840	0.074	0.186	0.165
**Kopner**									
r	− 0.096	0.124	− 0.073	0.384**	− 0.022	1	0.067	0.255*	− 0.163
*p* value	0.384	0.259	0.506	0.000	0.840	–	0.545	0.018	0.259
**Distri**									
r	− 0.046	0.081	− 0.233*	0.087	− 0.195	0.067	1	0.421**	− 0.035
*p* value	0.678	0.460	0.032	0.430	0.074	0.545	–	0.000	0.807
**VASI**									
r	0.089	0.308**	− 0.143	0.310**	0.145	0.255*	0.421**	1	− 0.016
*p* value	0.419	0.004	0.193	0.004	0.186	0.018	0.000	–	0.914
**VIDA**									
r	− 0.107	0.032	− 0.076	− 0.155	− 0.199	− 0.163	− 0.035	− 0.016	1
*p* value	0.458	0.823	0.599	0.282	0.165	0.259	0.807	0.914	–

Spearman’s Correlation coefficient are presented for different clinical characteristics among vitiligo patients’ understudy. Significant values are highlighted. **Correlation is significant at the 0.01 level (2-tailed). *Correlation is significant at the 0.05 level (2-tailed). *Distri.* distribution, Pre_Gray: pre greying, *VASI* vitiligo area severity index, *VIDA* vitiligo disease activity score.

### Pathway and function enrichment analysis for the circulating miRNAs in vitiligo

**Figure 3:**
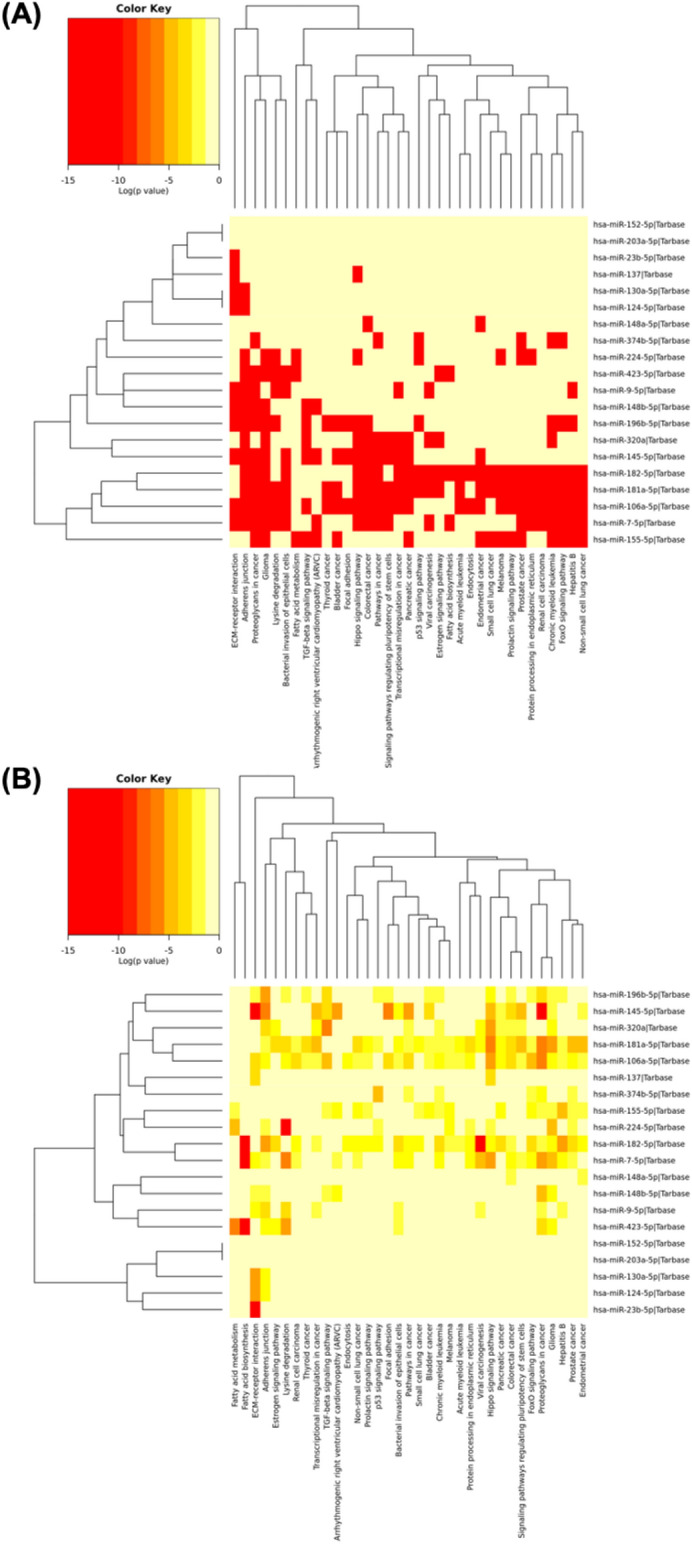
KEGG pathways enriched analysis for differentially expressed circulating miRNAs under study in vitiligo (**A**) Using targeted pathways clusters/heatmap. (**B**) Using significance clusters/heatmap.

A pathway enrichment analysis based on annotated gene targets in GO was performed to identify all the pathways targeted by DEmiRNAs in vitiligo. Different databases were used to assess the 20 miRNAs understudy regulatory functions and identify the miRNAs' molecular pathways under study. Enrichment of specific pathways revealed that the top pathways involved were proteoglycans in cancer, Hippo signaling pathway, fatty acid metabolism, protein processing in the endoplasmic reticulum, adherens junction, endocytosis, fatty acid biosynthesis, and biosynthesis of unsaturated fatty acids were found as shown in Fig. 3A,B.

**Figure 4:**
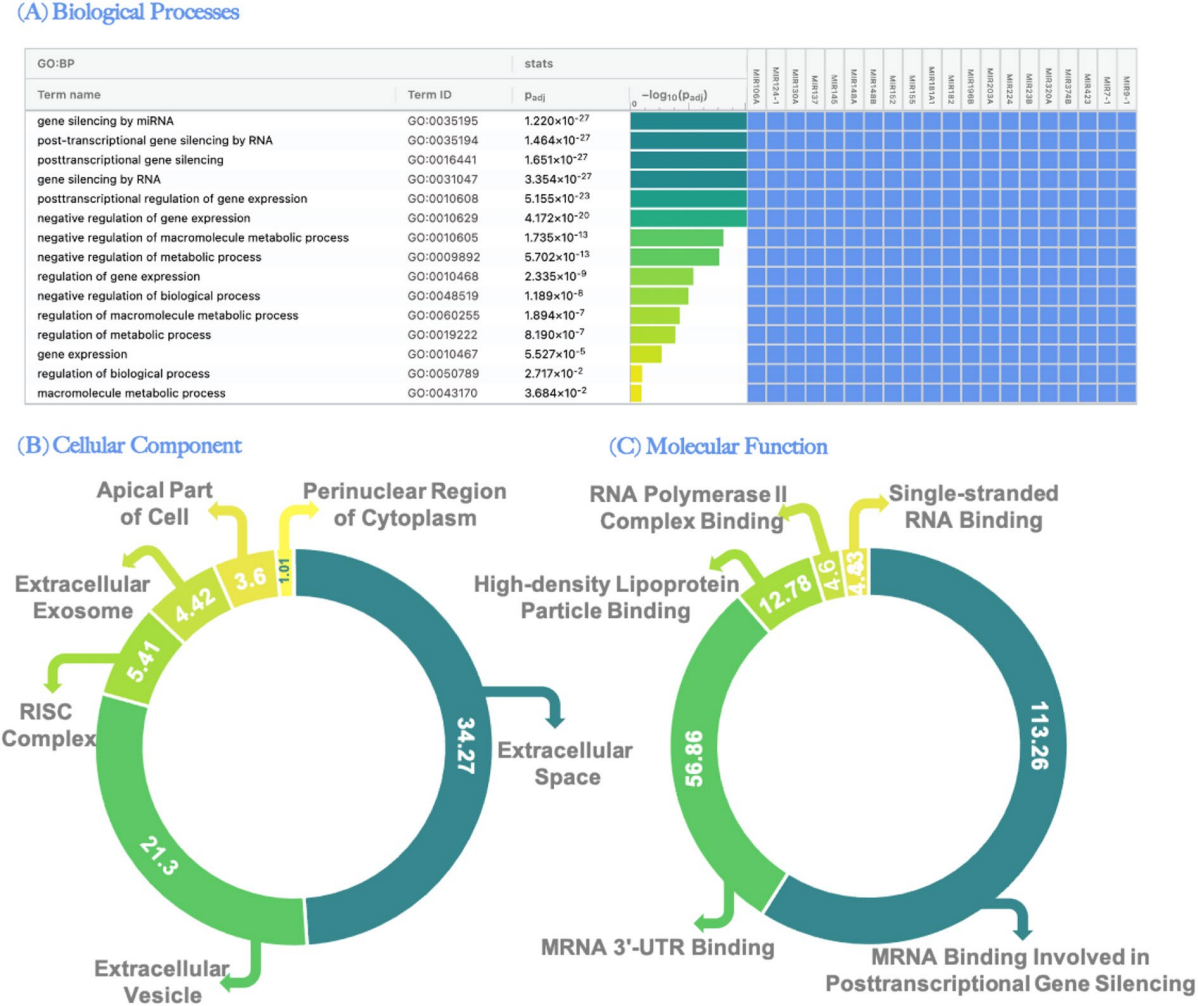
The gene ontology (GO) for the 20 circulating miRNAs under study. (**A**) Biological processes in descending order according to score detected using (http://biit.cs.ut.ee/gprofiler/gost) (**B**) Cellular component according to matching score detected using (https://geneanalytics.genecards.org) (**C**) Molecular function according to matching score detected using (https://geneanalytics.genecards.org).

The GO biological processes related to vitiligo pathogenesis were found to be distinctly enriched in our analysis were gene silencing by miRNA, posttranscriptional gene silencing by RNA, posttranscriptional gene silencing, gene silencing by RNA, posttranscriptional regulation of gene expression, negative regulation of gene expression, negative regulation of macromolecule metabolic process, negative regulation of the metabolic process, regulation of gene expression, and negative regulation of biological processes as represented in Fig. 4A.

The 20 circulating miRNAs understudy showed cellular localization in extracellular space, extracellular vesicle, RNA-induced silencing complex (RISC), extracellular exosome, apical part of the cell, and perinuclear region of cytoplasm as shown in Fig. 4B. Considering the molecular functions for the 20 miRNAs shown in Fig. 4C, the functions encompassed mRNA binding involved in posttranscriptional gene silencing, mRNA 3UTR binding, high-density lipoprotein particle binding, RNA polymerase II complex binding, and single-stranded RNA binding.

### MiRNA‐mRNA regulatory network construction

**Figure 5:**
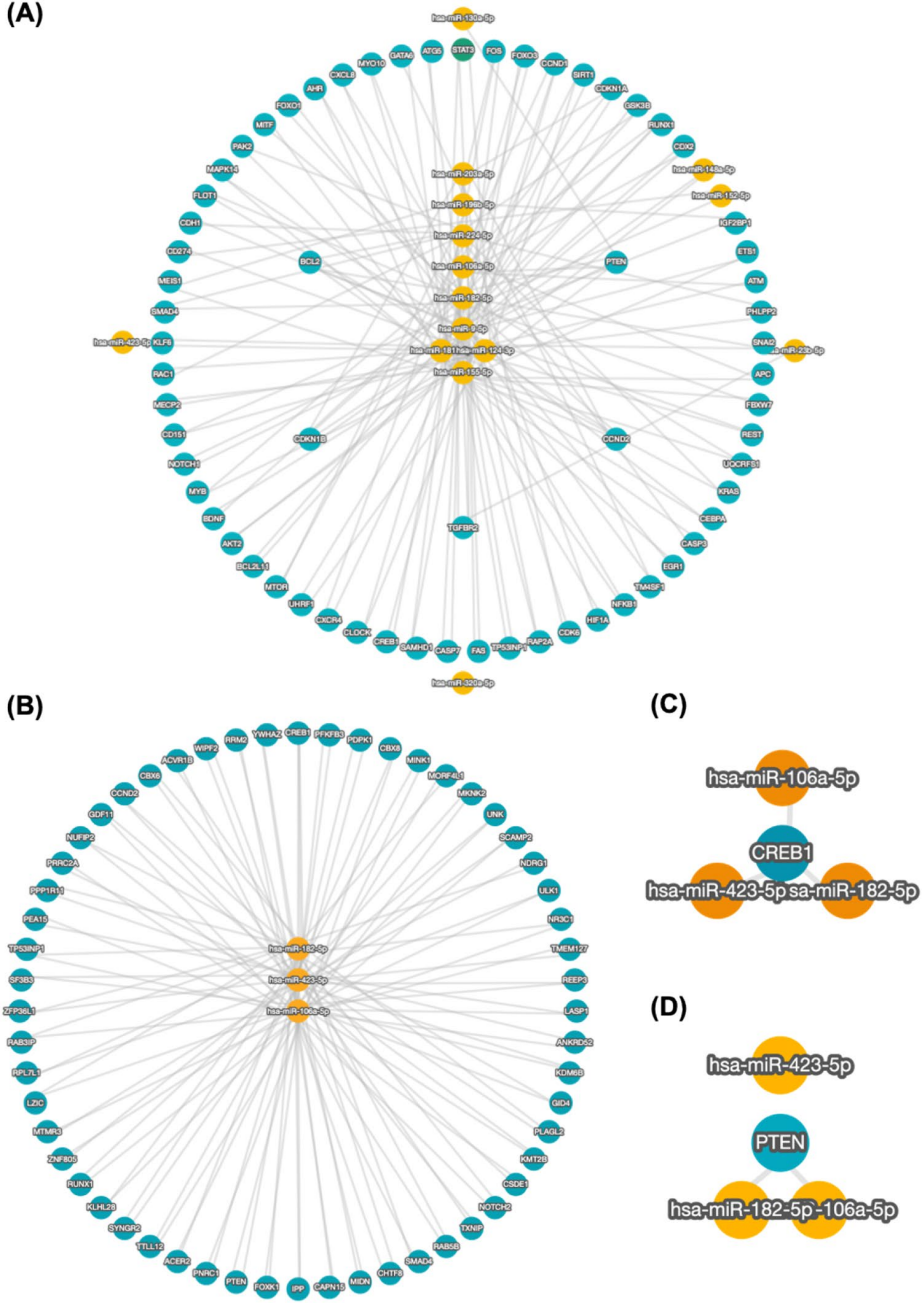
MiRNAs-target gene network analysis. (**A**) The miRNAs-target gene network with significant differential expression (16 out of 20) in our study. (**B**) The top three miRNAs according to ROC analysis and their target gene network. MiRNAs targets used were only the validated miRNAs whether with strong or weak validity with additional filter of minimum 2 shared targets. (**C**) The top three miRNAs according to ROC analysis with filtering options choosing strong validated targets only with minimum shared two targets revealing CREB1 gene as a common shared target (**D**) The top three miRNAs according to ROC analysis with filtering options choosing strong validated targets only with minimum shared three targets revealing PTEN gene as a common shared target for miR-182 and miR-106a but not with miR-432 using miRTargetLink 2.0 (https://ccb-compute.cs.uni-saarland.de/mirtargetlink2).

Our network analysis identified the relationship between the circulating miRNAs under study and their target genes. Our miRNA-target gene network comprised the 14 significant circulating miRNAs understudy presented in Fig. 5 revealing initially 186 target genes then filtered to include strong validated targets with a minimum of 2 shared targets that revealed a final of 65 target genes using miRTargetLink 2.0 (https://ccb-web.cs.uni-saarland.de/mirtargetlink/network.php) (Fig. 5A). The circulating miRNAs understudy and their targeted genes were related to the pathways potentially involved in vitiligo, such as fatty acid metabolism, protein processing in the endoplasmic reticulum, adherens junction, endocytosis, fatty acid biosynthesis, and biosynthesis of unsaturated fatty acids.

According to ROC analysis, the top three miRNAs that can be used as outstanding biomarkers in vitiligo, as depicted in Table 2, were miR-423, miR-106a, and miR-182. So, we have searched for their target gene network using strong and weak validated target with a minimum of 2 shared targets filter that revealed a final of 56 target genes using miRTargetLink 2.0 (https://ccb-web.cs.uni-saarland.de/mirtargetlink/network.php) (Fig. 5B). According to our study results, Kelch Like Family Member 28 (KLHL28), RAB3A Interacting Protein (RAB3IP), Notch Receptor 2 (NOTCH2), Phosphatase and Tensin Homolog (PTEN), CAMP Responsive Element Binding Protein 1 (CREB1), Midnolin (MIDN), Nuclear Receptor Subfamily 3 Group C Member 1 (NR3C1), Proliferation and Apoptosis Adaptor Protein 15 (PEA15), Ribonucleotide Reductase Regulatory Subunit M2 (RRM2), Tyrosine 3-Monooxygenase/Tryptophan 5-Monooxygenase Activation Protein Zeta (YWHAZ), Tumor Protein P53 Inducible Nuclear Protein 1 (TP53INP1), Forkhead Box K1 (FOXK1), and Cyclin D2 (CCND2) can be considered the critical genes involved in vitiligo in relation to miRNAs understudy. Further optimization of filtering options choosing strong validated targets only with minimum shared two targets revealed that CREB1 is a common shared target between the top three miRNAs by ROC analysis shown in Fig. 5C, while increasing shared targets to three revealed the PTEN as a common shared target for miR-182 and miR-106a but not with miR-432 (Fig. 5D).

## Discussion

In vitiligo, the search for non-invasive and reliable biomarkers for early diagnosis, prognosis, and treatment prediction is under intensive investigation. Although it is still a mystery how specific miRNAs regulate the melanogenesis pathway, in the current study, we identified a panel of miRNAs that could be used as potential biomarkers in vitiligo. Twelve out of the 20 circulating miRNAs showed significantly higher expression levels in vitiligo patients relative to controls where miR-423 show the highest expression level followed by miR-182, miR-106a, miR-155, miR-23b, miR-9, miR-124, miR-130a, miR-203a, miR-181, miR-152, and miR-320a. While four miRNAs (miR-224, miR-148a, miR-137, and miR-7) didn’t show significant expression level. On the other hand, four miRNAs (miR-148b, miR-145, miR-374b, and miR-196b) showed significantly lower expression levels in vitiligo patients relative to controls.

MiR-423 reported the highest over-expression level among the 20 miRNAs understudy with median log2 fold change (Q1 and Q3) of 5.7(4.06–7.3) and high discriminating power using ROC analysis (sensitivity = 97.6%, and specificity = 100%). MiR-423 has been reported to function as an oncomir in many cancers, including gastric cancer^27^, prostate cancer^28^, and glioblastoma^28^. In contrast, miR-423 functions as a tumor suppressor in osteosarcoma^29^ and ovarian cancer^30^. It was also initially identified as a circulating biomarker for heart disease, where Tijsen et al. reported overexpression of circulating miR-423 in patients with heart failure^31^. Other studies suggested that serum miR-423 plays a role in unstable angina pectoris^32^. Additionally, it was demonstrated that miR-423 is overexpressed in the plasma of pregnant women with preeclampsia^33^. Lastly, miR-423 showed to be a valid biomarker for active tuberculosis diagnosis^34^. Until now, miR-423 was investigated in very few studies related to non-cancerous skin diseases. Regarding vitiligo, Vaish et al. reported over-expression of miR-423 in lesional skin of vitiligo^35^, and Shang et al. showed an overexpression equivalent to 4.4-fold change using microarray with a significant *p* value in consistence with our results^15^. On the other hand, when Shang et al. validated their RT-PCR results, miR-423 showed non-significant results^15^.

The present study results showed novel findings correlating circulating miR-182, miR-106a, and miR-23b overexpression with vitiligo for the first time. MiR-182 was the second-highest over-expression level in our study with median log2 fold change (Q1 and Q3) of 5.1 (2.6–7.3) and outstanding discriminating power using ROC analysis. Although miR-182 was not previously correlated with vitiligo, it was extensively studied in melanoma. MiR-182 over-expression was reported in melanoma in many studies, and it was suggested to act via converging onto forkhead box O3 (FOXO3), melanocyte inducing transcription factor (MITF), reversion inducing cysteine rich protein with Kazal motifs (RECK), BCL2 apoptosis regulator (BCL2), and CCND2 inactivation or via epigenetic modulation of human melanoma cells^36–39^. These results suggest that miR-182 and its subsequent effect on vitiligo candidate genes such as MITF^40^ could be a useful prognostic or therapeutic biomarker in vitiligo.

Also, no previous results were reported on miR-106a differential expression role in vitiligo. In our study, miR-106a was the third-highest over-expression level with median log2 fold change (Q1 and Q3) of 4.5 (2.5–7.2) and outstanding discriminating power using ROC analysis. However, many studies reported the differential expression of miR-106a in melanoma^41–43^. Where they investigated its role as a tumor suppressor in melanoma by targeting E2F transcription factor 3 (E2F3)^41^. They also reported the role of its overexpression on melanoma cells via attenuating the effects caused by upregulating Connexin43 (Cx43) expression^42^ and its inhibition pro-oncogenic effect inhibition in melanoma cells in vitro^43^.

On the other hand, miR-106a was correlated with another autoimmune skin disorder, psoriasis. Miao et al. reported overexpression of serum level of miR-106a in psoriasis patients^44^, which was consistent with previous studies on psoriasis and autoimmune disorders^45,46^. Our study results provide new insight that miR-106a may have a role in vitiligo pathogenesis as it already plays a role in melanoma and psoriasis pathogenesis.

MiR-23b showed an overexpression level with median log2 fold change (Q1 and Q3) of 3.4 (1.3–5.8) and an excellent discriminating power using ROC analysis. No results were previously reported about its role in vitiligo. MiR-23b regulates normal physiological function, cellular immunity, and cell differentiation^47^ and has been shown to have a potent anti-inflammatory role in tissue-resident cells through its inhibitory actions on nuclear factor kappa B subunit 1 (NF-κB)^48,49^**.** Vellaichamy et al. reported underexpression of miR-23b in their in vivo model of post-inflammatory hyperpigmentation^50^, which is consistent with the downregulation of miR-23b reported in immune-mediated conditions such as lupus and rheumatoid arthritis^49^. Regarding melanoma, miR-23b was consistently downregulated in previous publications^51–53^ and showed that miR-23b targeted the Nicotinamide Phosphoribosyltransferase (NAMPT) gene^53^. MiR-23b was reported to have a significant role in autoimmune disorders via its downregulation by the effect of Interleukin 17A (IL-17A)^54,55^. Among autoimmune diseases, miR-23b proved to have a role in rheumatoid arthritis (RA), where Liu et al. and Abdeen et al. reported higher expression levels of miR-23b in RA patients^56,57^. Thus, elucidating the specific mechanism for miR-23b expression regulation can provide insights into its precise role in the pathogenesis of vitiligo.

MiR-155 was extensively studied in all autoimmune disorders, including vitiligo. In the present study, miR-155 showed an overexpression level with median log2 fold change (Q1 and Q3) of 3.8 (1.0–6.1) and high discriminating power using ROC analysis. Consistent with our results, Šahmatova et al. and Issa et al. reported overexpression of miR-155 in the epidermis and the peripheral blood of vitiligo patients, respectively^12,58^. Previous studies described the functions of miR-155 in vitiligo pathogenesis by targeting melanogenesis-associated genes such as tyrosinase related protein 1 (TYRP1), tyrosine 3-monooxygenase/tryptophan 5-monooxygenase activation protein epsilon (YWHAE), Syndecan Binding Protein (SDCBP), and SRY-Box transcription factor 10 (SOX10), thereby causing their inactivation^35^. In addition, the overexpression of miR-155 alters the levels of interferon-regulated genes such as interferon regulatory factor 1 (IRF1), suppressor of cytokine signaling 1 (SOCS1), and Interferon induced transmembrane protein 1 (IFITM1) in melanocytes and encourages interferon gamma (IFN-γ) and tumor necrosis factor alpha (TNF-α) expression confirming its role as proinflammatory miRNA^3,14,59^**.** Vaish et al. reported that miR-155 over-expression might contribute to vitiligo pathogenesis and recommended using an antagomiR for miR-155 and thus suppressing vitiligo progression^35^.

MiR-9 showed an overexpression level with median log2 fold change (Q1 and Q3) of 3.1 (0.9–5.1) and good discriminating power using ROC analysis. MiR-9 is considered one of the miRNAs associated with oxidative stress in vitiligo (Li et al.). MiR-9 expression is affected by oxidative stress and is responsible for mediating ROS pathogenic effect in vitiligo^60^**.** Sirtuin 1 (SIRT1) gene is known to be a target for miR-9^61^. Previous studies have indicated that SIRT1 protects against stress-related diseases by interacting with forkhead box protein (FOXO), NFκB, protein P53 (p53), and peroxisome proliferator-activated receptor-gamma coactivator 1-alpha (PGC-1α), which regulate various cellular processes, including inflammation and stress responses^62,63^. A recent Egyptian study by Raia et al. reported overexpression of miR-9 in both serum and tissues of vitiligo patients^64^. They also reported a statistical correlation between miR-9 and patients’ VASI scores^64^. However, Su et al., Shi et al. and Kadir et al. reported downregulation of miR-9 resulting from inducing Interleukin 10 (IL-10) involved in UVB mediated vitiligo repigmentation where IL-10 is known to be an essential immunoregulatory element that is decreased in vitiligo^3,65,66^.

MiR-124 was identified as a brain-enriched miRNA, but it is expressed in a wide range of human/animal tissues participating in the pathogenesis of several disorders^67^. MiR-124 plays different roles in various pathologic conditions and suppresses acute stress and inflammatory responses^68^. In the present study, miR-124 showed an overexpression level with median log2 fold change (Q1 and Q3) of 3.0 (0.3–5.4) and an excellent discriminating power using ROC analysis. It was not previously reported to have a role in vitiligo, but it was reported to be implicated in other autoimmune disorders. Regarding RA, the miR-124 level was underexpressed in RA tissues as reported by Nakamachi et al. and directly downregulating the production of CDK-2 and MCP-1^68^. In addition, Nakamachi et al. concluded that miR-124 might be a promising therapeutic agent for RA and other autoimmune diseases^68^. MiR-124 was also downregulated in different cancers^69^, and it functions as a tumor suppressor in melanoma^70^.

MiR-130a can function as either an oncogene or tumor suppressor in many human diseases^76^. In the present study, miR-130a showed an overexpression level with median log2 fold change (Q1 and Q3) of 2.76 (0.2–4.9) and a good discriminating power using ROC analysis. It has not been previously investigated in vitiligo, but few studies elucidated its role in cancer and autoimmune disease. A previous study on miR-130a revealed that it could stop cancer metastasis by enhancing antitumor host immunity^77^. Another study reported the oncogenic role of miR-130a in triggering tumor growth and malignant cell survival by targeting phosphatase and tensin homolog^78^. At the same time, Wu et al. indicated that miR-130a acts as a tumor suppressor to reduce uveal melanoma metastasis by activating the Wnt/β-catenin signaling pathway by targeting the oncogene Ubiquitin Specific Peptidase 6 (USP6)^79^. From the perspective of autoimmune diseases, Wade et al. reported an overexpression level of miR-130a in psoriatic patients with higher disease activity^80^. Wade et al. also stated that miR-130a was among the highest accuracy miRNAs in the stratification of patient response that distinguished good/moderate and non-responders in early psoriatic arthritis^80^.

In the present study, miR-203a showed an overexpression level with median log2 fold change (Q1 and Q3) of 2.4 (0.24–5.0) and a fair discriminating power using ROC analysis. MiR-203a is a skin-specific miRNA, significantly expressed only in differentiated suprabasal keratinocytes by asymmetric cell division via a driven transcriptional activation mechanism^81^. MiR-203a has been reported to target significant pigmentation genes like Tyrosinase (TYR)^82,83^, MITF^84^, SRY-Box Transcription Factor 9 (SOX9), TYRP1, RAB27a, Myosin VA (MYO5a), and Fascin Actin-Bundling Protein 1 (FSCN1)^59^. MiR-203 role in vitiligo has been investigated in very few studies, where it did not reveal conclusive results^12^. Šahmatova et al. results were in contrast to ours as they did not detect any differences in miR-203a expression levels in the skin of control subjects and patients with vitiligo^12^. In response to narrowband ultraviolet B (NB-UVB), the classical pathway of the p53/αMSH/MC1R/MITF cascade is activated in the keratinocytes while Transforming Growth Factor Beta 1 (TGF-β1) ligand secretion via the miR-203/c-Jun pathway is suppressed, which stimulates the differentiation and proliferation of melanocytes^85^. NB-UVB also stimulated heparan sulfate-binding growth factors via activating heparanase release, which facilitate the melanogenesis process^86,87^. MiR-203a overexpression has a critical role in psoriasis pathogenesis^13,85,88,89^ as it has a role in regulating the keratinocyte differentiation and proliferation as it directly targets and negatively regulates several epidermal genes^90^. Further, miR-203 with its known function as a key regulator of epidermal differentiation, has been shown to hinder stemness by decreasing the expression of the p63 and thus, affecting melanosome transport mechanisms and subsequently melanogenesis^91^.

MiR-181a showed overexpression in the present study with median log2 fold change (Q1 and Q3) of 2.4 (− 0.04 to 4.3) and adequate discriminating power using ROC analysis. MiR-181a has not been previously overexpressed in vitiligo, but it has been reported as a central metabolic regulator during development and homeostasis^92^. It has been implicated in various diseases^93–95^. Among its essential targets is the PTEN gene, where the overexpression of miR-181a acts as the PTEN gene suppressor and is associated with a variety of solid tumors, leukemias, and obesity^93–95^. So, it is currently considered one of the principal guardians of cellular and metabolic regulation^96^.

MiR-148a, miR-148b, and miR-152 represent members of the miR-148/-152 family^97^. In the present study, miR-148a and miR-152 were significantly differentially expressed with median Log2 fold change (Q1 and Q3) of 1.2 (− 1.2 to 3.5) and 2.0 (−0.08 to 3.8), respectively. At the same time, miR-148b did not show a significant expression level in vitiligo patients compared to controls. These three miRNAs were not previously investigated in vitiligo. The miR-148/-152 family plays a crucial role in different physiologic and pathological processes, including tumorigenesis and autoimmune diseases. The expression of these miRNA family members is regulated by hypermethylation, transcription factors, cytokines, long non-coding RNAs (lncRNAs), and signal transduction pathways. Their differential expression pattern in autoimmune diseases and tumors suggests that they might be used as prognostic biomarkers and/or as therapeutic targets for these diseases^97^.

MiR-320a was significantly overexpressed in vitiligo patients than normal controls with median log2 fold change (Q1 and Q3) of 1.6 (− 0.3 to 3.3) in the present study. No previous studies reported a differential expression level of miR-320a in vitiligo. MiR-320a is a member of the miRNA320 family explicitly expressed in epithelial tissues and is involved in skin development, functional maintenance, and homeostasis; thus, it is an essential regulatory factor in the skin^98^. A study by Wei et al. found that miR-320 expression is significantly underexpressed in psoriatic lesions than in healthy control skin tissues; miR-320 is postulated to participate in psoriasis development by regulating surviving gene expression^98^. MiR-320 also serves as a potential biomarker for glucose and lipid metabolism-associated diseases like diabetes, atherosclerosis, adiposity, and nonalcoholic fatty liver disease^99^.

MiR-224 showed significant expression level in vitiligo patients compared to normal controls with median log2 fold change (Q1 and Q3) of 1.59 (− 0.5 to 3.8) in the present study. MiR-224 is differentially expressed in biological processes, including cell proliferation, migration, and invasion, in various malignancies^100–104^. In consistence with our results, Wang et al. reported significant overexpression of miR-224 in the peripheral blood mononuclear cells (PBMC) patients with non-segmental vitiligo. Thus, they suggested that specific miRNAs signatures in PBMC are a part of the vitiligo-associated immune response, and miRNA may serve as novel drug targets for vitiligo therapy^14^. Also, Rashed et al. reported overexpression of miR-224 in lesional skin of vitiligo compared to normal controls^105^.

MiR-137 regulates melanocyte differentiation by repressing MITF expression^71^ and exerts antitumor effects in melanoma cells by regulating MITF, c-MET, Y-box-binding protein 1, and enhancer of zeste homolog 2 (EZH2)^72^. In the present study, miR-137 showed non-significant underexpression with median log2 fold change (Q1 and Q3) of 0.9 (− 1.6 to 2.7). In contrast to our results, Dong et al. found overexpression of miR-137 in their study that targets c-KIT in melanocytes^73^. Overexpression of miR-137 may reduce the expression of Tyrosinase Related Protein 2 (TYRP2) and c-KIT and reduce the increase in melanin production caused by ultraviolet (UV) treatment^74^. It is suggested that miR-137 can inhibit melanogenesis in mouse skin melanocytes by inhibiting the expression of c-KIT and TYRP2 in the SCF/c-KIT signaling pathway^75^. Interestingly, miR-137 showed an effect on coat color, demonstrating that changes in the expression of a specific miRNA may significantly affect melanogenesis; however, till now, this is evident only in animal models^73^.

Different studies revealed that miR-145 plays a role in melanogenesis via targeting genes that are essential for melanogenesis, including Rho Associated Coiled-Coil Containing Protein Kinase 1 (ROCK1), Eukaryotic Translation Initiation Factor 2 Alpha Kinase 1 (EIF2AK1), and Calcium/Calmodulin Dependent Protein Kinase ID (CAMK1D) and the pigmentation process as MITF, TYR, and TYRP1^17,61,106^, our results reported non-significant differential expression of miR-145 which was underexpressed in our study. In contrast to our results, Issa et al. reported overexpression of miR-145 in the peripheral blood of vitligo patients compared to controls^58^. Another study also described a decrease in cell proliferation and initiation of apoptosis via caspase-3 and caspase-7 induction due to miR-145 overexpression in non-lesional skin of patients with vitiligo via targeting ROCK1, CAMK1D, and EIF2AK1^61,106^. Furthermore, Dyndoot et al. reported underexpression of miR-145 in cultured pigment cells after induction of pigmentation^59^**.** In line with our results, Vaish et al.^35^ reported that miR-145 was downexpressed in vitiligo lesional skin.

Although previous studies reported overexpression of miR-196b in patients with vitiligo, miR-196b showed non-significant underexpression levels in our study^15^. Moreover, polymorphisms involving miR-196 were suggested to play a significant role in the pathogenesis and the prognosis of vitiligo^15^.

In conclusion, to better understand the molecular mechanism of vitiligo pathogenesis, it is fundamental to have more information regarding miRNAs and their functions. However, little is known about miRNA-based regulations in vitiligo pathogenesis. This study is the first Egyptian study to analyze the melanogenesis pathway miRNAs-related expression profile in the plasma of patients with NSV. The data raise the possibility that miRNAs may be involved in the pathogenesis of NSV. Based on these results, we suggest that specific circulating miRNAs signature might be a part of the immune response implicated in vitiligo pathogenesis, and miRNAs could potentially be used as biomarkers for skin pigmentation disorders, including vitiligo.

Further multicentric studies with a larger number of cases are needed to emphasize whether the dysregulated miRNAs described in the present study can be utilized as diagnostic, prognostic, and/or therapeutic markers and/or targets for vitiligo. Finally, as there was an overlap in the miRNAs signature pinpointed from different studies, we recommend more observational and experimental research on the studied miRNAs. Observational studies should try to standardize the laboratory methods and address larger cohorts to elucidate miRNAs' role in vitiligo pathogenesis. Experimental studies, whether in vitro or in vivo animal models, should investigate the effect on the melanogenesis process followed by functional and clinical validation studies using miRNA inhibitors or mimetics.

## Data Availability

The datasets used and/or analyzed during the current study are available from the corresponding author on reasonable request.

## Abbreviations

3′UTR: 3′ Untranslated region
AUC: Area under the curve
BCL2: BCL2 apoptosis regulator
CAMK1D: Calcium/calmodulin dependent protein kinase ID
CCND2: Cyclin D2
CREB1: CAMP responsive element binding protein 1
Cx43: Connexin43
DAVID: Database for Annotation Visualization and Integrated Discovery
DEmiRNAs: Differentially expressed miRNAs
Distri.: Distribution
DM: Diabetes mellitus
E2F3: E2F transcription factor 3
EDTA: Ethylene diamine tetra-acetic acid
EIF2AK1: Eukaryotic translation initiation factor 2 alpha kinase 1
EZH2: Enhancer of zeste homolog 2
FC: Fold change
FH: Family history
FOXK1: Forkhead box K1
FOXO: Forkhead box protein
FOXO3: Forkhead box O3
FSCN1: Fascin actin-bundling protein 1
GO: Gene ontology
HTN: Hypertension
IFITM1: Interferon induced transmembrane protein 1
IFN-γ: Interferon gamma
IL-10: Interleukin 10
IL-17A: Interleukin 17A
IRF1: Interferon regulatory factor 1
KEGG: Encyclopedia of genes and genomes
KLHL28: Kelch like family member 28
LncRNAs: Long non-coding RNAs
MIDN: Midnolin
MIQE: Minimum information for publication of quantitative real-time PCR experiments
miRNAs: MicroRNAs
MITF: Melanocyte inducing transcription factor
MYO5a: Myosin VA
NAMPT: Nicotinamide phosphoribosyltransferase
NB-UVB: Narrowband ultraviolet B
ncRNAs: Non-coding RNAs
NF-κB: Nuclear factor kappa B subunit 1
NOTCH2: Notch receptor 2
NR3C1: Nuclear receptor subfamily 3 group C member 1
NSV: Non-segmental vitiligo
P53: Protein P53
PBMC: Peripheral blood mononuclear cells
PEA15: Proliferation and apoptosis adaptor protein 15
PGC-1α: Peroxisome Proliferator-activated receptor-gamma coactivator 1-alpha
Pre_Gray: Pre greying
PTEN: Phosphatase and tensin homolog
QoL: Quality of life
qRT-PCR: Quantitative real-time PCR
RA: Rheumatoid arthritis
RAB3IP: RAB3A interacting protein
RECK: Reversion inducing cysteine rich protein with Kazal motifs
RISC: RNA-induced silencing complex
ROC: Receiver operating curve
ROCK1: Rho associated coiled-coil containing protein kinase 1
RRM2: Ribonucleotide reductase regulatory subunit M2
RT: Reverse transcription
SCU: Suez Canal University
SDCBP: Syndecan binding protein
SIRT1: Sirtuin 1
SOCS1: Suppressor of cytokine signaling 1
SOX9: SRY-box transcription factor 9
SOX10: SRY-box transcription factor 10
SPSS: Statistical package for the social sciences
SV: Segmental vitiligo
TGF-β1: Transforming growth factor beta 1
TNF-α: Tumor necrosis factor alpha
TP53INP1: Tumor protein P53 inducible nuclear protein 1
TYR: Tyrosinase
TYRP1: Tyrosinase related protein 1
TYRP2: Tyrosinase related protein 2
USP6: Ubiquitin specific peptidase 6
UV: Ultraviolet
VADI: Vitiligo disease activity score
VASI: Vitiligo area severity index
VETF: Vitiligo European Task Force
YWHAE: Tyrosine 3-monooxygenase/tryptophan 5-monooxygenase activation protein epsilon
YWHAZ: Tyrosine 3-monooxygenase/tryptophan 5-monooxygenase activation protein zeta
